# Analysis of colorectal cancers in British Bangladeshi identifies early onset, frequent mucinous histotype and a high prevalence of *RBFOX1* deletion

**DOI:** 10.1186/1476-4598-12-1

**Published:** 2013-01-03

**Authors:** Neel Sengupta, Christopher Yau, Anuratha Sakthianandeswaren, Dmitri Mouradov, Peter Gibbs, Nirosha Suraweera, Jean-Baptiste Cazier, Guadalupe Polanco-Echeverry, Anil Ghosh, Mohamed Thaha, Shafi Ahmed, Roger Feakins, David Propper, Sina Dorudi, Oliver Sieber, Andrew Silver, Cecilia Lai

**Affiliations:** 1 Centre for Digestive Diseases, Blizard Institute of Cell and Molecular Science, Barts and The London School of Medicine and Dentistry, Queen Mary University of London, 4 Newark St, Whitechapel, London, E1 2AT, UK; 2 Department of Mathematics, South Kensington Campus, Imperial College London, London, SW7 2AZ, UK; 3 Ludwig Colon Cancer Initiative Laboratory, Ludwig Institute for Cancer Research, Parkville, VIC, 3050, Australia; 4 Bioinformatics and Statistical Genetics, Wellcome Trust Centre for Human Genetics, Roosevelt Drive, Oxford, OX3 7BN, UK; 5 Present address: Genomic Services, Wellcome Trust Centre for Human Genetics, Roosevelt Drive, Oxford, OX3 7BN, UK; 6 Academic Surgical Unit, The Royal London Hospital, 80 Newark St, Whitechapel, London, E1 2ES, UK; 7 Department of Pathology, The Royal London Hospital, 80 Newark St, Whitechapel, London, E1 2ES, UK; 8 St Bartholomew’s Hospital, Gloucester House, Little Britain, London, UK; 9 Faculty of Medicine, Dentistry and Health Sciences, Department of Surgery, University of Melbourne, Parkville, VIC, 3050, Australia

**Keywords:** Colorectal cancer, Genome analysis, British Bangladeshi, *RBFOX1*, *KRAS*, *BRAF*

## Abstract

**Background:**

Prevalence of colorectal cancer (CRC) in the British Bangladeshi population (BAN) is low compared to British Caucasians (CAU). Genetic background may influence mutations and disease features.

**Methods:**

We characterized the clinicopathological features of BAN CRCs and interrogated their genomes using mutation profiling and high-density single nucleotide polymorphism (SNP) arrays and compared findings to CAU CRCs.

**Results:**

Age of onset of BAN CRC was significantly lower than for CAU patients (p=3.0 x 10^-5^) and this difference was not due to Lynch syndrome or the polyposis syndromes. *KRAS* mutations in BAN microsatellite stable (MSS) CRCs were comparatively rare (5.4%) compared to CAU MSS CRCs (25%; p=0.04), which correlates with the high percentage of mucinous histotype observed (31%) in the BAN samples. No *BRAF* mutations was seen in our BAN MSS CRCs (CAU CRCs, 12%; p=0.08). Array data revealed similar patterns of gains (chromosome 7 and 8q), losses (8p, 17p and 18q) and LOH (4q, 17p and 18q) in BAN and CAU CRCs. A small deletion on chromosome 16p13.2 involving the alternative splicing factor *RBFOX1* only was found in significantly more BAN (50%) than CAU CRCs (15%) cases (p=0.04). Focal deletions targeting the 5’ end of the gene were also identified. Novel *RBFOX1* mutations were found in CRC cell lines and tumours; mRNA and protein expression was reduced in tumours.

**Conclusions:**

*KRAS* mutations were rare in BAN MSS CRC and a mucinous histotype common. Loss of *RBFOX1* may explain the anomalous splicing activity associated with CRC.

## Background

Colorectal cancer (CRC) is common [1] and most cases arise sporadically. A small proportion of CRC is associated with inherited syndromes such as familial adenomatous polyposis (FAP; <1% of CRC), MUTYH-associated polyposis (MAP; rare recessive condition, carrier estimated at ~1%) and Lynch syndrome / hereditary non-polyposis colon cancer (LS/HNPCC; 2-4% of CRC) [1]. FAP patients have germline mutations in one allele of the adenomatous polyposis (*APC*) tumour suppressor gene, MAP patients carry biallelic mutations in the *MUTYH* oxidative damage repair gene and LS is caused by germline mutations in DNA mismatch repair genes (mostly *MLH1* and *MSH2,* less commonly *MSH6* and *PMS2*) [1]. Clinically, LS differs from FAP in that CRC arises without extensive polyposis. CRC in FAP, MAP and LS are characterised by an early age of onset (FAP, mean age, 40 years [2,3]; MAP, median, 45 years (range, 21–67) [4]; LS, median 43 years (range, 20–79) [5]). In comparison, sporadic CRC is rare until the sixth decade [6].

Sporadic CRCs are defined by their dominant genomic instability pathway and classified into two major groups. Chromosome unstable CRC (CIN) are characterised by gross chromosomal abnormalities and present in approximately 65-70% of CRC [7]. Microsatellite unstable (MSI) CRCs account for another 15%, and are driven by defective mismatch repair, which causes increased mutations of short nucleotide repeats (microsatellites) [8]. These two genomic instability pathways are associated with a difference in prognosis: MSI cancers behave less aggressively [9]. However, the classification of sporadic CRCs as CIN or MSI is not always straightforward, as a significant proportion appears microsatellite and chromosome stable (MACS) [10,11]. The frequent presence of epigenomic instability in sporadic CRC in the form of global hypomethylation or the CpG island methylator phenotype further complicates the picture [11,12].

Over a third of the population in the borough of Tower Hamlets, London, United Kingdom (UK) are Bangladeshi in origin. Interestingly, despite a high incidence of diabetes mellitus in the BAN population group [13], prevalence of CRC is lower at 27/100,000 compared to 342/100,000 in the non-Bangladeshi British population [14]. In an expression array profiling study of a small cohort of BAN CRCs, Ahmed et al. [14] reported that the percentage of cases aged less than 40 was high (61%).

The reduced susceptibility to CRC in the BAN population and the apparent early age of CRC onset are intriguing. Here, we compared the clinicopathological characteristics of BAN CRC patients presenting to our local hospital to those from a group of sporadic CAU CRC patients. We compared molecular genetic profiles between tumours from these patient groups, including genome-wide DNA copy number alterations, *KRAS* and *BRAF* mutation to test whether CRCs in the BAN population showed molecular features distinct from CAU CRCs.

## Results

### Clinicopathological features of patients

Between June 1997 and August 2008, all patients with CRC were invited to participate in research and CRCs were resected from 44 consented BAN patients at our centre. BAN CRC patients were recruited sequentially with patients from the CAU population and there was no prior selection; no BAN patients were missed during this time period. The age of CRC onset in the BAN cohort (median, 58; range, 25–80) was significantly lower (p=3.0 × 10^-5^) than for sporadic CAU CRC patients (median 71; range, 21–91) presenting at the same hospital. Although the early age of onset could be partly attributed to a relatively young population [15], it can also be an indication of a higher prevalence of inherited CRC syndromes or other predisposing conditions. However, as none of the BAN CRC patients in our cohort had a history of polyposis or chronic gastrointestinal inflammation when they were diagnosed, it is unlikely that their cancers are associated with FAP, MAP or inflammatory bowel disease.

The lower age of onset might reflect a higher prevalence of LS. To investigate this, we performed genotyping with MSI markers BAT25 and BAT26 using DNA extracted from tumour and matched normal tissue samples available from 37 patients (age of onset ≤45, n=17; age of onset >45, n=20; median age, 51). We identified 7 patients (4 early-onset, 3 late-onset) with MSI (all positive at both the BAT25 and BAT26 loci). In addition, immunohistochemistry detected loss of one or more mismatch repair proteins (MLH1, MLH6 or PMS2) in five of the seven cases (Additional file 1: Table S1). No tumour showed loss of MSH2. Two of these patients showed an early-onset of cancer at 40 and 43 years of age whereas the remaining three were older at diagnosis (65, 71 and 73). Hence, genetic features suggestive of potential LS cases were detected in 5 out of 37 patients in our cohort (13.5%), and in 2 out of 17 patients belonging to the early age group (11.8%). Because family histories were usually not available from the BAN patients, supporting evidence for a familial predisposition to CRC could not be obtained. However, additional cancers presented in two of the five potential LS cases: gastric adenocarcinoma in one individual and both an adenocarcinoma of the lung and endometrial cancer in another (Additional file 1: Table S1). In order to obtain a more uniform group of sporadic BAN samples for further investigation, we excluded all seven patients whose tumours were MSI and an additional patient (age, 51 years) whose tumour showed loss of PMS2 protein expression. Importantly, this did not alter the median age of onset (51.6 vs. 51.4 years). The clinicopathological data on these BAN patients with sporadic MSS stable cancers are summarised in Table 1.

**Table 1:** Clinical and pathological characteristics of the BAN MSS colorectal cancers

	**Overall**	**Early onset (≤45 years)**	**Late onset (>45 years)**	**P value**
	**(n = 29)**	**(n = 13)**	**(n = 16)**	**(Early Vs Late)**
*Age of Onset - Yr*				
Median	57	35	64	*-*
Range	25-80	25-45	51-80	
*Sex – No (%)*				
Female	10 (34.5)	5 (38.5)	5 (31.3)	N.S.
Male	19 (65.5)	8 (61.5)	11 (68.7)	
*Site of Cancer – No (%)*				
Right	6 (20.7)	0 (0)	6 (38.5)	0.021
Left	23 (79.3)	13 (100)	10 (62.5)	
*Dukes’ Stage – No (%)*				
A	3 (10.3)	1 (7.7)	2 (12.5)	N.S.
B	6 (20.7)	2 (15.4)	4 (25)	
C	19 (65.5)	10 (76.9)	9 (56.2)	
D	1 (3.4)	0 (0)	1 (6.3)	
*Mucin - No (%)*				
No	20 (69.0)	7 (53.8)	13 (81.2)	N.S.
Yes	9 (31.0)	6 (46.2)	3 (18.8)	
*Vascular Invasion - No (%)*				
No	16 (55.2)	8 (61.5)	8 (50)	N.S.
Yes	13 (44.8)	5 (38.5)	8 (50)	
*Differentiation - No (%)*				
Well	1 (3.4)	1 (7.7)	0 (0)	N.S.
Moderate	24 (82.8)	10 (76.9)	14 (87.5)	
Poor	4 (13.8)	2 (15.4)	2 (12.5)	
*Resection Status - No (%)*				
0	21 (72.4)	10 (76.9)	11 (68.7)	N.S.
1	7 (24.1)	3 (23.1)	4 (25)	
2	1 (3.4)	0 (0)	1 (6.3)	

All p values were calculated using Fisher’s Exact Test (two-tailed) and p ≤ 0.05 was considered to be significant.

Subsequent comparisons focused on the remaining 29 BAN MSS patients with presence of mismatch repair protein expression: 13 patients diagnosed under the age of 45 were classified as early-onset (median, 34; range, 25–45), while the remaining 16 patients diagnosed over the age of 45 (median, 64; range, 51–80) were classified as late-onset. Overall, BAN MSS cancers were frequently left-sided (79.3%) and more prevalent in males (65.5%). Most of the cancers were moderately differentiated (82.8%). There was a high percentage of mucinous tumours (31%) compared to the background CAU population (5-15%) [16]. Within the BAN MSS group, left-sided location was significantly more prevalent in early-onset (13/13, 100%) than in late-onset cancers (10/16, 63%) (p=0.02, Fisher’s Exact Test) (Table 1). However, early-onset and late-onset BAN MSS cancers had similar male: female ratios and showed no difference in stage, prevalence of mucinous phenotype, frequency of vascular invasion, degree of differentiation or resection (R) status (Table 1).

### Sequence analysis of *KRAS* and *BRAF* mutation hotspots

We compared the mutation frequencies of *KRAS* and *BRAF* between BAN CRC and CAU CRCs by performing sequence analysis of known mutation hotspots (codon 12 and 13 for *KRAS* and codon 600 for *BRAF*). In the BAN MSS samples (n=29) we found two mutations at codon 12 of *KRAS* (2/29, 5.4%), one in an early-onset and another in a late-onset CRC. This mutation rate is lower than in our previously characterised CAU MSS CRCs (*KRAS*, 33/134, 25%) [11], and the differences between populations were significant (p=0.04). Interestingly, the presence of *KRAS* mutations has been associated with a non-mucinous histotype [17]. The low prevalence of *KRAS* mutations in our BAN MSS samples may be correlated to the high percentage of mucinous tumour observed (30% in BAN MSS samples with wildtype *KRAS*). No mutations were observed in the *BRAF* gene in BAN. Although a lower frequency of *BRAF* mutation is expected for MSS CRCs, this frequency is still lower than that observed in our CAU MSS sample set (17/144, 12%).

### Genomewide copy number analysis using single nucleotide polymorphism (SNP) beadarray

The pattern of somatic genomic copy number alterations in BAN and CAU CRCs was evaluated using SNP arrays. DNA was obtained from ten matched tumour-normal pairs from the BAN MSS patient group (early-onset, n=4; late-onset, n=6), where fresh frozen tissues were available. For comparison, we extracted DNA from paired tumour and normal tissues from a group of MSS CRC from the CAU population (n=27). We had previously classified these samples as CIN (n=11) and MACS (n=16) CRCs, using flow cytometry ploidy analysis and loss of heterozgosity (LOH) markers specific to chromosome instability [11].

In total, 37-paired CRC samples were hybridised onto Illumina bead arrays (BAN, n=10, Hap550 bead arrays; CIN, n=11, and MACS, n=16, HumanHap370- Duo bead arrays) and analysed using OncoSNP [18]. The use of high-density SNP array allowed a wide range of genomic alterations to be determined, including small amplifications, small deletions, and copy number neutral loss of heterozygosity (2nLOH) events. All tumour samples contained genomic alterations. To obtain an overview of the genetic complexity of the samples using SNP array data, we determined: (i) the average ploidy of the sample, by taking the average copy number over all SNPs; (ii) the percentage of the genome altered, by taking the fraction of SNPs showing somatic differences compared to the matched normal. In order to support our analysis, we also used genome-wide SNP genotyping data from an additional dataset generated from 636 CRC samples to provide a background for visualisation.

**Figure 1:**
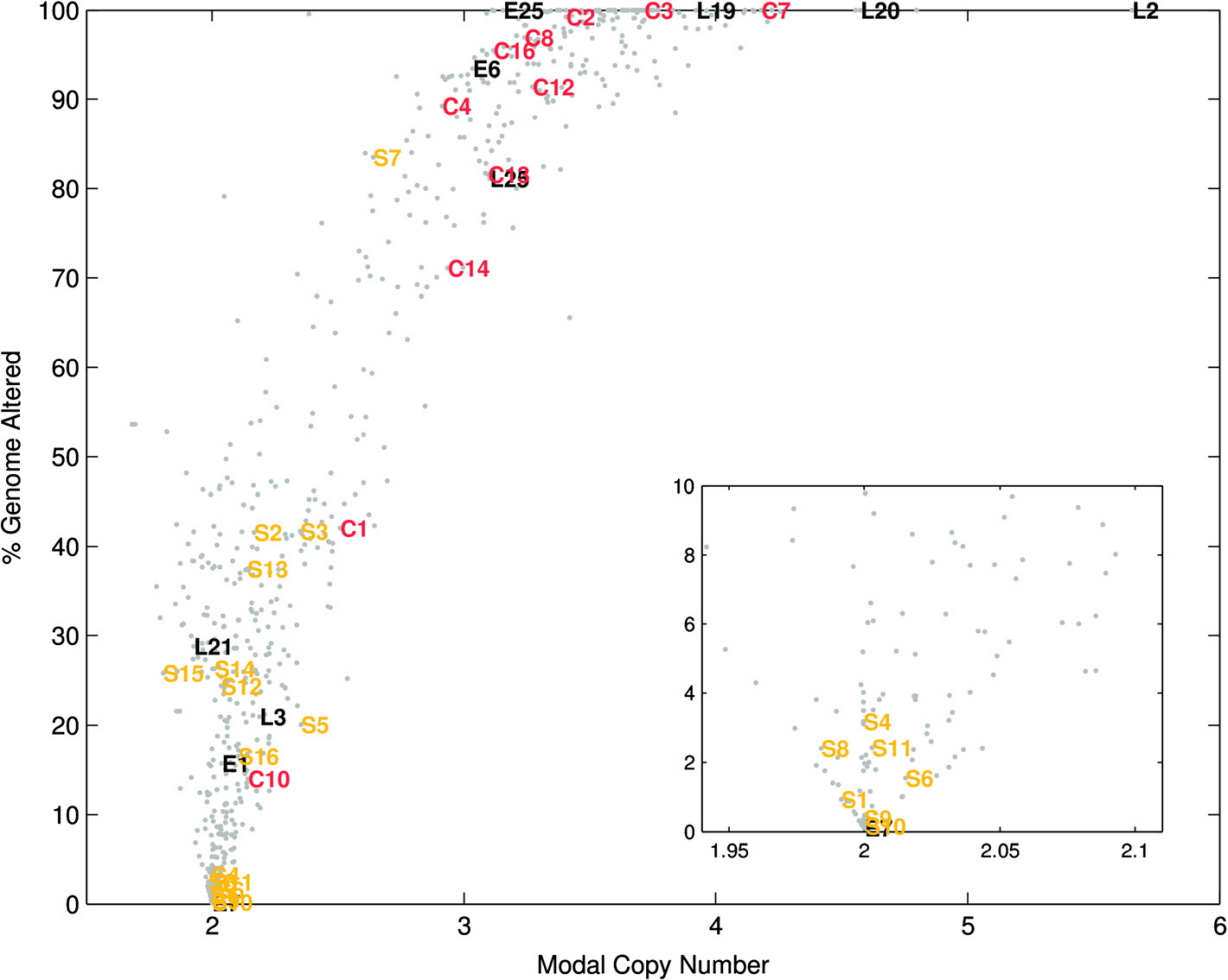
**The distribution of genomic complexity for (C)IN, MAC(S), (E)arly and (L)ate-onset colorectal cancer.** Data for 636 samples (grey) from the Ludwig Colon Cancer Initiative data set was used as background reference. (Inset) Expanded view showing clustering of samples with few genomic aberrations.

The cancer samples fell into three broad, distinct clusters (Figure 1). The first was a diploid group (average ploidy=2) of cancers with a minimal number of genomic aberrations (<10%). This group contained seven of the sixteen MACS samples (44%) and one of the four early-onset cancers (25%) from the BAN cohort. By definition, we would not expect any CIN cancers to be associated with this group, and our SNP genotyping data supported this. The second was the set of chromosomally unstable cancers (>50% genome altered) exhibiting polyploidy (average ploidy >3). As expected, the majority of the CIN cancers, as defined by earlier ploidy analysis (82%, 9/11), was located in this group, and was accompanied by a single MACS (6%) CRC. Also in this set were two early-onset (50%) and four late-onset BAN cancers (67%). A third intermediate group (Figure 1) had an average ploidy >2 and moderate levels of genomic aberrations (10-50%). Within this group there was a concentration of MACS cancers (50%, 8/16) and early- (25%, 1/4) and late-onset (33%, 2/6) BAN MSS cancers along with a small number of CAU CIN CRCs (18%, 2/11)

Genome-wide permutation testing was used to assess if any SNPs were significant markers for differentiating between early- and late-onset BAN cancers. In our sample set, no individual marker or set of markers had any stratification effect (p >0.001).

### Chromosomal regions frequently altered in Bangladeshi and Caucasian colorectal cancers

**Figure 2:**
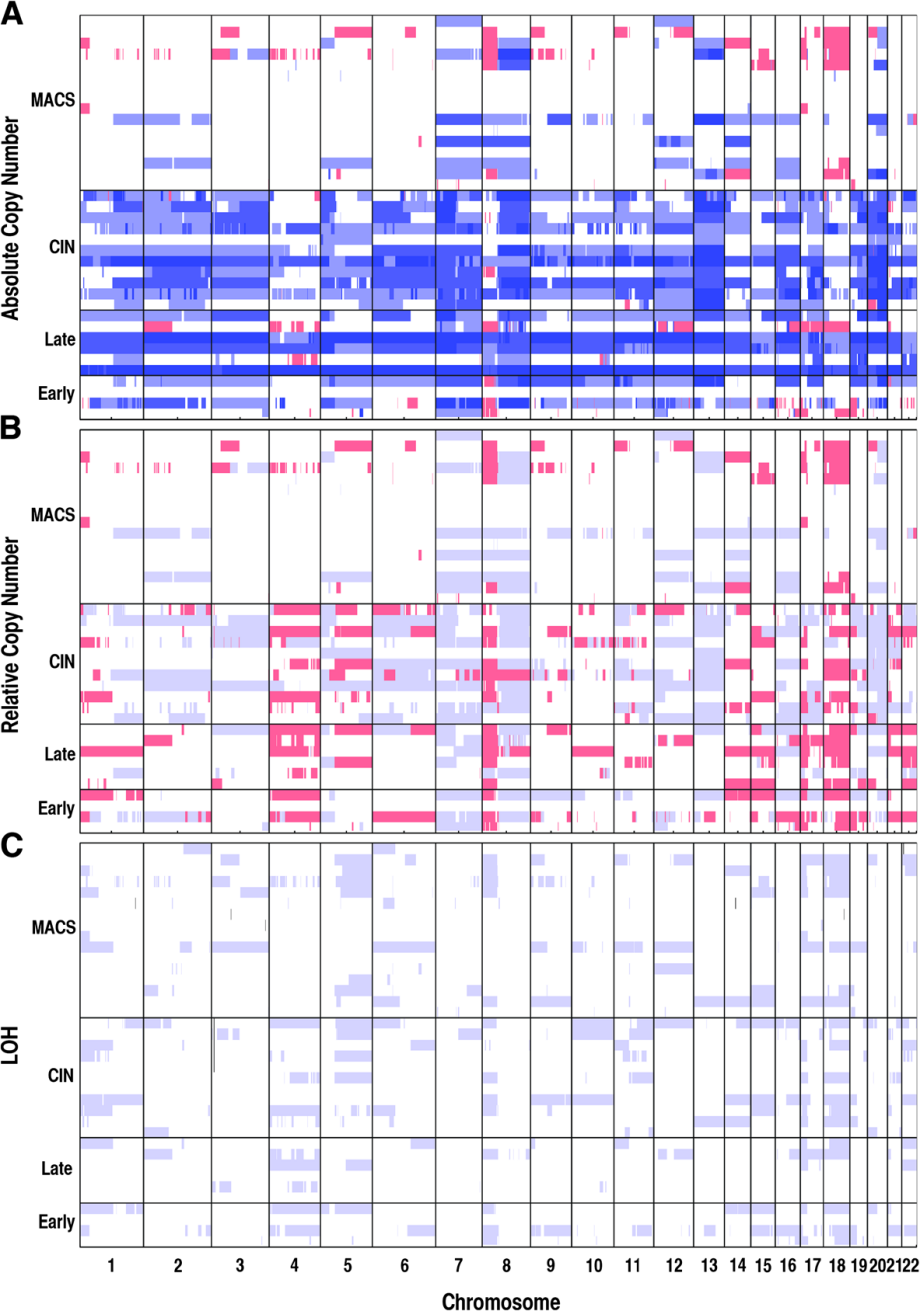
**Genome-wide copy number profiles. (A)** Genome-wide absolute copy number profiles for the four classes of colorectal cancers. Blue – Copy number gain (>2). Red – Copy number loss (<2). **(B)** Genome-wide relative (to average ploidy) copy number profiles. Blue – relative gain, Red – relative loss. **(C)** Genome-wide LOH profiles.

In terms of absolute copy number change (Figure 2A, in blue), the most frequently observed gene amplifications in the BAN CRC samples were located on chromosomes 7, 8q and 17q. This pattern overlaps with CAU MSS cancers where amplification in CIN CRCs are mostly observed on chromosomes 2q, 5p, 7p, 13, 17q and 20q, and in MACS cancers on chromosomes 7, 8q and 20q (Figure 2A). After eliminating amplification due to increased ploidy, the relative copy number gains in the BAN samples remained on chromosomes 7 and 8q (Figure 2B). Absolute copy number loss (Figure 2A, in red) was relatively rare compared to gains, with the most noticeable losses in the BAN CRCs located on chromosome 8p.

A different picture emerged when deletions relative to average ploidy were analysed (Figure 2B, in red), and regions of frequent relative loss in BAN samples included chromosomes 8p, 17p and 18q (8/10, 80%). These results, again, showed similarities with the alterations detected typically in the CIN and MACS CAU cancers, although few regions of loss were detected in MACs samples (Figure 2B). In the BAN samples, LOH was not observed as frequently as amplification and deletions (Figure 2C), and the regions with the highest frequency of LOH were located on 4q, 17p and 18q (5/10, 50%). The pattern of LOH in BAN samples was again similar to that observed in the CIN and MACS sporadic cancers, where LOH was observed repeatedly on chromosomes 17p (13/27, 48%) and 18q (15/27, 55%).

In total, over 45% of our cases showed deletions and/or LOH at chromosome 17p (BAN: 5/10, 50%; CIN: 5/11, 45%; MACS: 7/16, 44%). The genomic regions altered in most cases were large, encompassing the majority of the short arm of chromosome 17. Importantly, almost all of these alterations involve LOH or allelic deletion at the *TP53* locus (17p13.1) (BAN, 4/5, 80%; CIN, 5/5, 100%; MACS, 6/7, 86%). Loss of *TP53* is a consistent feature across the BAN, CIN and MACS CRCs and is therefore likely to be the major factor influencing tumour development at this locus. A similar situation was observed on chromosome 18q, where *SMAD4* (18q21.2) was lost in six BAN samples (6/10; 60%) six CIN samples (6/11, 55%) and three MACS samples (3/16; 19%). In comparison, gross chromosomal alterations at the *APC* locus (5q22.2) were less frequent in all subgroups of samples (BAN, 3/10, 30%; CIN, 4/11, 36%; MACS, 3/16, 19%).

### A small deletion on chromosome 16p13.2, containing the *RBFOX1* gene, was detected in both Bangladeshi and Caucasian CRCs

**Figure 3:**
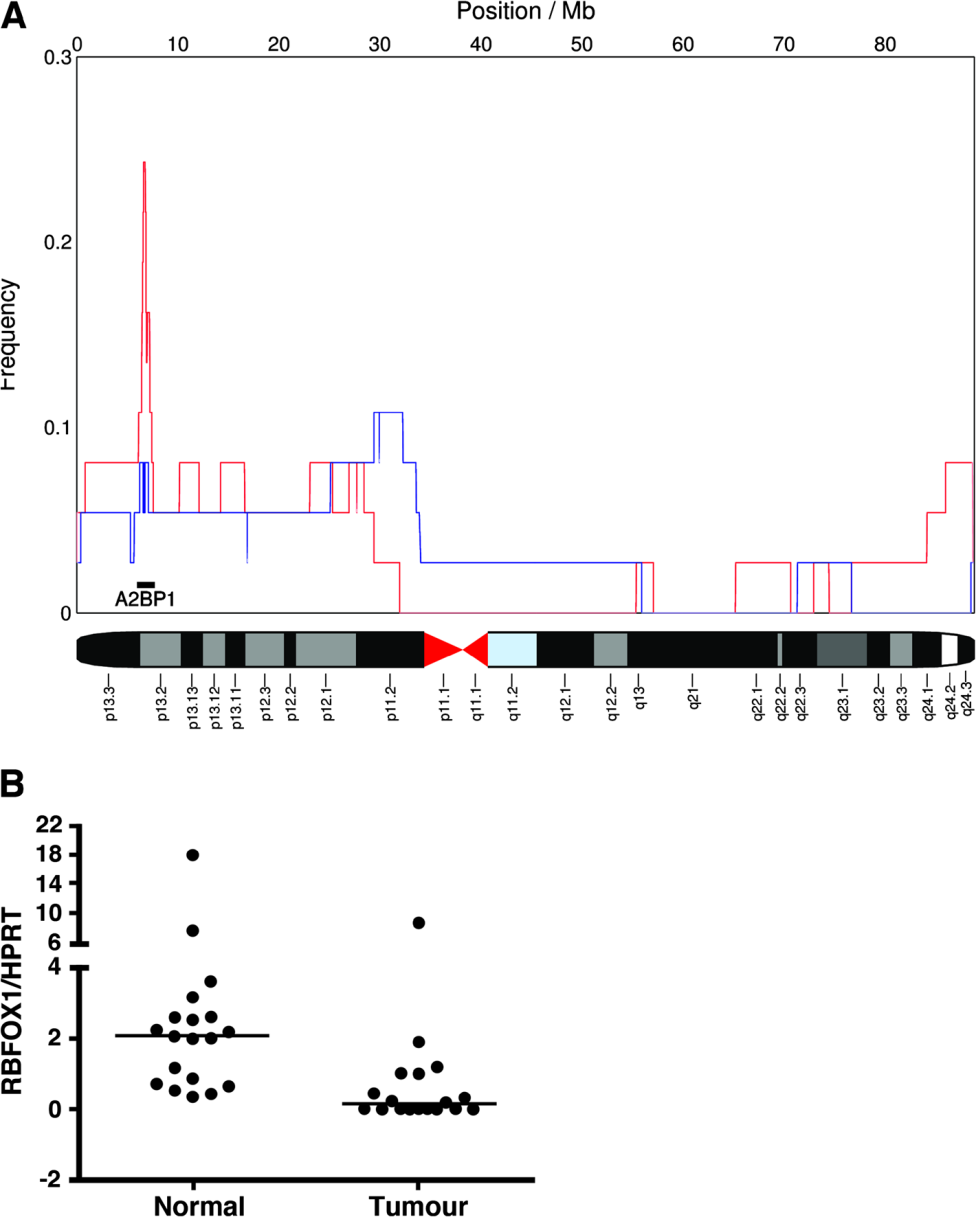
**Genomic alterations and expression changes of *RBFOX1* in CRC. (A)** Analysis of relative copy number (blue) gain and (red) loss across all cancers (CIN, MACS and BAN) shows recurrent deletions on chromosome 16p13 affecting the *RBFOX1* gene. **(B)** Scatter plot showing *RBFOX1* gene expression level as detected by quantitative real time PCR. Nineteen tumour / normal sample pairs were tested and mRNA level is expressed as ratio of *RBFOX1* and *HPRT* concentration, [*RBFOX1*] / [*HPRT*].

Interestingly, we detected a small deletion on chromosome 16p13.2 from 6.0 to 7.7 Mb in ten samples (Figure 3A), including five BAN (5/10, 50%), 2 from early-onset and 3 from late-onset, three CIN (3/11, 27%) and one MACS (1/16, 6%). Accordingly, this deletion might be more frequent in BAN CRCs than CAU CRCs (5/10 versus 4/27; p=0.04, Fisher’s Exact Test). According to the University of California Santa Cruz (UCSC) Genome Bioinformatics database (http://genome.ucsc.edu/), this genomic region contains only one known gene, RNA binding protein, fox-1 homolog (*RBFOX1*). Deletion of *RBFOX1* has been detected in 35% of MSI and 21% of MSS colon tumours in a previous study, and fluorescence *in situ* hybridization was performed to confirm the somatic deletion of this locus in four samples [19]. Deletion of *RBFOX1* was also presented in a significant proportion of CRC (106/419) in The Cancer Genome Atlas dataset [20] (Additional file 2: Figure S1). The deletion clusters included hemizygous loss of the entire gene and more focal deletions primarily targeting the regulatory and 5’ exons of *RBFOX1*.

**Figure 4:**
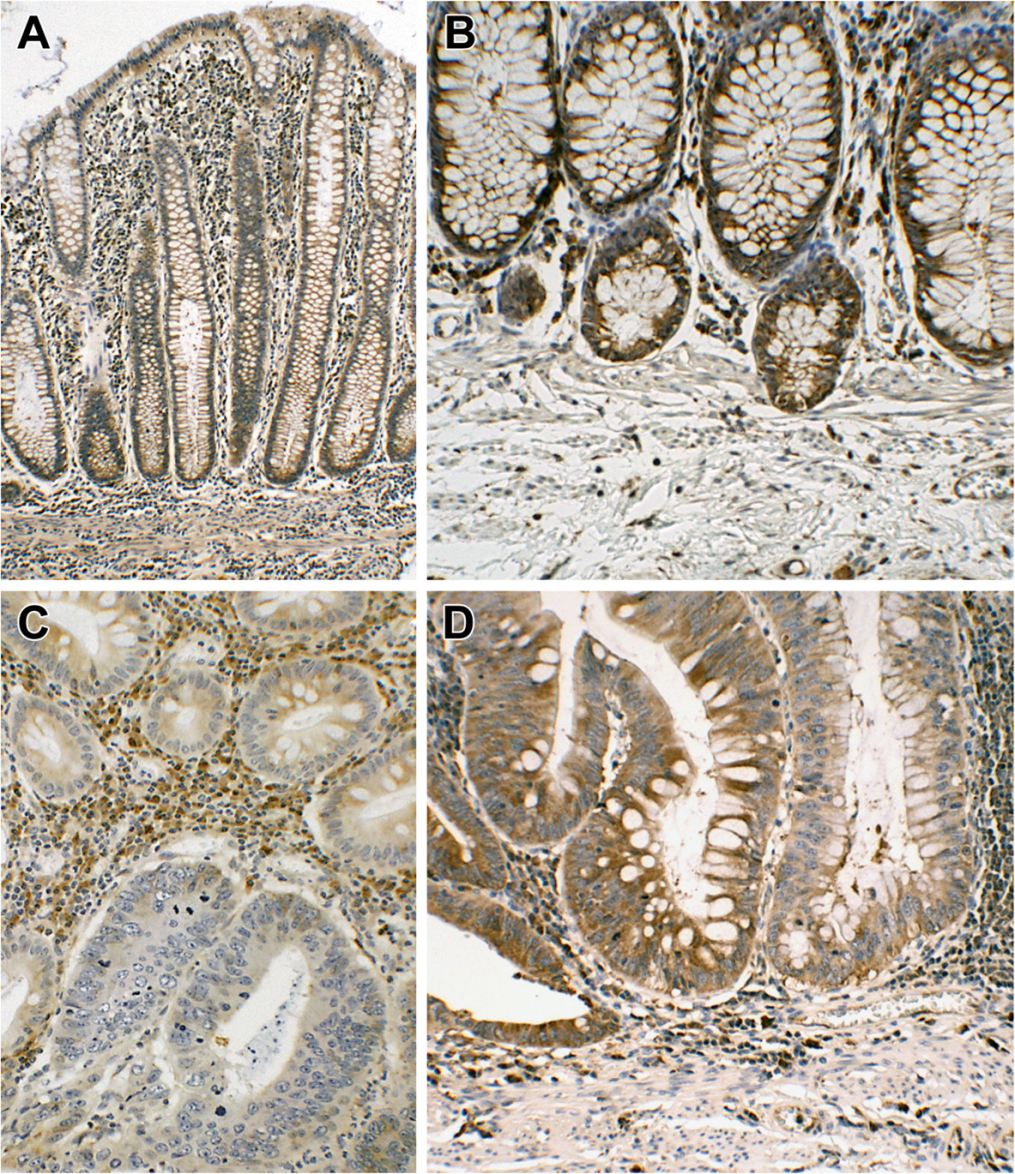
**Immunohistochemical detection of RBFOX1. (A)** Normal appearing colonic mucosa with diffuse cytoplasmic staining of most epithelium (x100). **(B)** Normal colon with diffuse staining of the epithelium and some stromal cells and inflammatory cells; the pericryptal sheath fibroblasts appear unstained (x200). **(C)** Adenocarcinoma showing absence of RBFOX1 staining in the majority of the cells lining the two-neoplastic glands in the lower half of the picture (x200). **(D)** Neighbouring glands show persistence (left) or reduction/ loss (right) of RBFOX1 immunoreactivity on epithelial cell cytoplasm (x200).

*RBFOX1* is an RNA binding protein, which has a role in splicing regulation, and the gene is transcribed into a few alternatively spliced isoforms [21,22]. Although *RBFOX1* is highly expressed in the nucleus and / or cytoplasm in the brain, heart and skeletal muscle, its expression in the intestines has not been defined clearly. Therefore, we sought evidence of *RBFOX1* expression by quantitative real time PCR (QRT-PCR) using cDNA from 43 colorectal cell lines and 19 paired CAU CRC tumour/normal tissues, where good quality RNA was available. In most cell lines (36/43, 84%), the threshold cycle (Ct) values produced in the QRT-PCR experiment indicated absence or undetectable levels of *RBFOX1* mRNA transcript, whereas low level of *RBFOX1* mRNA was found in the remaining seven cell lines (Ct range: 33.1-33.8) (Additional file 3: Table S2). In contrast, *RBFOX1* mRNA was detected in all normal tissues samples (Ct range: 23.6-36.7, median, 31.6). Furthermore, in most tumour samples the expression level of *RBFOX1* was reduced compared to the normal paired tissue samples (Figure 3B, Additional file 3: Table S2). As *RBFOX1* appears to be expressed at low level, the possibility that its expression is restricted to particular cell types was considered. The immunohistochemical expression of RBFOX1 in human non-neoplastic colon and colon cancer tissues was assessed (Figure 4). Staining was detected in the cytoplasm of normal colonic epithelium and reduced levels of RBFOX1 expression were observed in the some of the tumour samples we tested (Figure 4C).

To assess the mutation frequency of *RBFOX1* in CRC, we performed high-resolution melt (HRM) analysis on 39 CRC cell lines and 137 tumour and normal samples. All changes were then confirmed by bidirectional Sanger sequencing. In total, we found six novel mutations in five cell lines, (5/39, 12.8%) and three novel mutations in two tumour/normal samples pairs (2/133, 1.5%) (Table 2, Additional file 4: Table S3). The somatic mutations were further analyzed for the potential or likely effect of amino acid substitutions on protein structure and function, using Sorts Intolerant From Tolerant (SIFT) and Polymorphism Phenotyping (PolyPhen-2) (Additional file 4: Table S3). Of the nine novel mutations, two were predicted to be possibly damaging/deleterious by SIFT and PolyPhen-2, two were silent mutations and the remaining five produced contradictory predictions by SIFT and PolyPhen-2. Further investigations will be required to understand the functional effects of these somatic mutations on *RBFOX1* function and CRC.

**Table 2:** ***RBFOX1 *mutations and single nucleotide polymorphisms found in CRC cell lines and patient tumour samples**

	**Mutations**	**Known SNPs**
**Cell Lines**		
CoCM1	c.776C>T; p.Ser259Phe	
HCT15	c.288C>T; p.Ser96Ser	
LIM2405		c.393A>G; p.Glu131Glu (rs140174146)
LIM2551	c.154delC	
LOVO	1. c.293C>T; p.Ala98Val	
	2. c.353C>T; p.Thr118Met	
LS411	c.293C>T; p.Ala98Val	
**Patient Samples**		
33	1. c.851C>T; p.Ala284Val	
	2. c.1023G>A;	
	p.Gln341Gln	
53	c.350C>T; p.Pro117Leu*	
93	c.598G>A; p.Val200Met	

*Mutation from patient 53 is identical to known SNP rs139251660, but was present only in tumour but not in the paired normal tissue.

## Discussions

Very little research has been done to characterise CRCs arising in Bangladeshi patients, despite the reportedly low frequency of CRC and suggestions of a high proportion of early-onset CRCs in this population [14]. Here we confirmed that BAN patients have a relatively early age of onset of CRC compared to CAU patients presenting at the same hospital and that the age of onset remains low even after potential LS cases have been omitted. This raises the possibility that BAN CRCs have arisen as a result of environmental influences and/or a genetic predisposition that can lead to somatic molecular changes and clinicopathological features dissimilar to those found in tumours from the wider UK population. This study aimed to uncover any clinical or genetic features specific to BAN CRCs.

We observed no major differences in the clinical features between early- and late-onset BAN MSS, and most findings were consistent with CAU MSS cancers reported previously [11]. Survival of the early-onset and the late-onset BAN MSS CRC was similar, even though the early-onset cases were all left-sided unlike the late-onset (62.5% left-sided). We cannot, however, rule out a relationship between age of onset and survival as our cohort was relatively small. It also remains to be seen if BAN CRC characteristics change over time and between generations, particularly as environmental factors such as diet change can impact on the population.

There were, however, an unexpectedly large proportion of mucinous tumours in the BAN sample set. This observation may be linked to the reduced frequency of *KRAS* mutations in BAN MSS compared with CAU CRCs and CRCs in other populations. Specifically, *KRAS* is mutated at frequencies between 30%-44% in African American, Chinese and Japanese CRCs [23-25]. It is difficult to ascertain if the high proportion of early-onset cases causes the reduction in the number of *KRAS* mutations in BAN, as previous studies suggested only marginal differences between early-onset and late-onset CAU CRCs with regard to *KRAS* mutations (<50 years, 29%; >50 Years, 33%) [26]. These frequencies also concur with those we reported previously for CAU CRCs [11]. On the other hand, there is evidence that *KRAS* mutation frequency is lower in early-onset CRC in India (≤50 years, 24%; ≥60 Years, 47%) [27]. Further investigation in BAN CRCs will help to explain the aetiology behind the low *KRAS* mutations and high mucin percentage.

We found no *BRAF* mutations in BAN MSS CRC, although the absence of MSI cancers in the cohort may contribute to this observation [28]. Overall, it will be interesting to confirm these results in a larger cohort of BAN patients and establish how this may affect clinical decision, particularly in view of the increased use of targeted therapy in CRC.

We utilised high-density SNP bead arrays to detect any genomic alterations that might be specific to BAN patients. The BAN cohort contained both diploid and polyploid cancers, and the copy number changes detected were largely comparable to those seen in the CAU CRCs. We also identified small, heterozygous deletions on chromosome 16p13.2 in a substantial number of BAN and CAU MSS CRCs. Deletion of this part of the genome has been previously described in a number of studies on genomic alterations in CAU and Japanese CRCs [29-33].

In our sample set, the deletion was more frequent in BAN CRCs compared to CAU cancers, although there was no difference in occurrence between early- and late-onset BAN CRCs. This region of the genome encodes *RBFOX1*, a highly conserved RNA-binding protein that regulates tissue-specific alternative splicing indicating important basic functions in development and differentiation. When we examined *RBFOX1* in a large number of CRCs from The Cancer Genome Atlas Network sample set, we found that the regulatory regions and 5’ exons of the gene is often deleted, which may lead to aberrant *RBFOX1* expression or isoform distribution. Interestingly, in a recent study the 5’ untranslated region of *RBFOX1* was also rearranged in 4/25 melanoma [34], pointing to the possible importance of this part of the gene.

Very little is known about the expression or role of *RBFOX1* in the intestine. Using immunohistochemistry, we confirmed that RBFOX1 is expressed at low levels in normal gut tissues and that expression is often lost in CRC. We also identified a small number of novel somatic mutations in CRC. Therefore, *RBFOX1* appears to be targeted by various mechanisms in CRC. Functionally, loss of RBFOX1 activity may lead to aberrations in the splicing of a significant number of genes, generating diverse functional products that vary from those found in normal tissue [35]. Alternative splicing is a key feature of cancer [36-38] including CRC [35,39] and the identification of *RBFOX1* targets in CRC will help to determine if *RBFOX1* deletion is a critical feature of dysfunctional splicing in CRC.

To our knowledge, this is the first report of genomic characterisation of CRC in British Bangladeshi populations. We concluded that there were no genetic copy number alterations unique to BAN MSS CRCs, but that mutations of RAS signalling oncogenes were comparatively rare. The functional role of *RBFOX1* mutations in BAN and CAU MSS CRC highlights aberrant alternative splicing in CRC as an important mechanism for further study.

## Materials and methods

### Patients and tumour samples

CRCs were resected from 44 patients of Bangladeshi origin at Barts and the London NHS Trust between January 1997 and August 2008. Tumour tissue (fresh frozen or formalin fixed paraffin embedded) was obtained from 37 patients. Within this group, 17 (46%) were 45 years old or under at the time of diagnosis.

Fresh-frozen tumor and matched normal tissues were retrieved from 133 stage II and III CRC patients treated at the Royal Melbourne Hospital and Western Hospital Footscray, Australia. A total of 67 individuals were female and 66 were male, with a median age at diagnosis of 73 years (range, 30–92 years). 89 cancers were stage II and 44 were stage III. 57 cancers were from the proximal colon, 64 from the distal colon and 12 from the rectum.

### Microsatellite instability genotyping and immunohistochemistry of mismatch repair protein

For microsatellite instability assay, DNA was extracted from tumour tissues using QIAamp DNA mini kit (Qiagen, Hilden, Germany) and genotyping was performed using BAT25 and BAT26 mononucleotide markers [40] on the ABI 3100 DNA Sequencer (Applied Biosystems) and analysed using GeneScan software (Applied Biosystems). For fresh frozen tumour tissues, only samples with >80% tumour were used in experiments, and FFPE tumour tissues were macrodissected before DNA extraction. Tumours were classified as microsatellite unstable if one or more of the markers showed bandshift of 3 base pairs or more. The expression of the mismatch repair proteins was assessed using immunohistochemistry (IHC) [41].

### Statistical analysis

GraphPad Prism version 4.03 for Windows (GraphPad Software, San Diego, California, USA) was used for statistical analysis.

### Single nucleotide polymorphism beadarrays and data analysis

Single nucleotide polymorphism (SNP) genotyping of Bangladeshi and Caucasian MSS CRCs was performed using the HumanHap 550 Duo and 370 Duo BeadChip (Illumina) respectively, after confirmation of DNA quality using Agilent biochips. For comparison, we also utilised Ilumina 610-Quad SNP genotyping data from 636 cancers obtained from the Ludwig Colon Cancer Initiative, Australia.

For copy number analysis, SNP data for the tumour samples were pre-processed by regressing Log R Ratio values for the tumour against corresponding values for the paired normal to reduce the effect of array artefacts. The SNP data was then examined for copy number and LOH changes in paired tumour/normal tissue using OncoSNP (version 2.25) [PMID: PMC2965384]. We used the intra-tumour heterogeneity option with 10 EM iterations and sub-sampling window size of 30. Two early-onset BAN and 4 CIN samples were excluded on the basis of poor data quality. For comparisons, copy number and LOH calls from the Illumina Human Hap500 and 610-Quad SNP genotyping data were remapped using nearest neighbour interpolation on to the HumanHap370-Duo probe set. *FOXRB1* deletions were further investigated in a dataset of 419 colorectal cancers from The Cancer Genome Atlas Project. Affymetrix SNP 6.0 CEL files were preprocessed and converted into the OncoSNP (v1.3) format using the PennCNV-Affy tool [PMID: PMC2045149]. Colorectal tumours showing *RBFOX1* deletions were identified and then clustered using a k-means algorithm. Genomic coordinates are in Human Genome Build hg18.

### Mutation analysis

Mutation hotspots in *KRAS* and *BRAF* were analysed by direct sequencing (Life Technologies). *RBFOX1* exons were screened using high resolution melt curve analysis on xthe ABI 7500 Fast Real Time PCR system (Life Technologies) and analysed using the HRMv2.0.1 software (Life Technologies). Somatic mutations were then validated by bidirectional Sanger sequencing twice and confirmed to be absent in paired normal tissues. Primer sequences are available from authors. Mutations were analysed for the effect of amino acid substitutions on protein structure and function, using Sorts Intolerant From Tolerant (SIFT) at http://sift.bii.a-star.edu.sg/ and Polymorphism Phenotyping (PolyPhen-2) at http://coot.embl.de/PolyPhen/.

### Quantitative real time PCR and immunohistochemistry on *RBFOX1*

Quantitative Real-time polymerase chain reaction (QPCR) was performed using TaqMan assays (Applied Biosystems) against *RBFOX1* (Hs00251554_m1) and thecontrol housekeeping gene HPRT (hypoxanthine phosphoribosyltransferase 1;HuHPRT_0604009). Immunohistochemistry was carried out using rabbit polyclonal primary antibody against RBFOX1 (sc-135476, Santa Cruz Biotechnology).

### Ethics approval

Ethical approval for this study was obtained from the Human Research Ethics Committees of all sites and all patients gave informed consent.

## Competing interest

The author(s) declare that they have no competing interests.

## Authors’ contributions

NSe, ASa, NSu, GPE, CL performed the experiments and data analysis. CY, DM, JBC carried out bioinformatics and biostatistics. PG, AG, MT, SA, RF, DP, SD provided tissue samples and clinical data. RF performed analysis for immunohistochemistry. OS, ASi and CL contributed to design the studies, interpretion of the data and wrote the manuscript. All authors read and approved the final manuscript.

## Supplementary Material

Additional file 1 **Table S1.** Loss of mismatch repair proteins MLH1, MLH6 and PMS2 in a subset of British Bangladeshis colorectal cancers. No tumour showed loss of MSH2. Click here for file

Additional file 2 **Figure S1.** UCSC Genome Browser showing hemizygous deletions/LOH (Red) and homozygous deletions (Yellow) of *RBFOX1* in The Cancer Genome Atlas colorectal adenocarcinoma sample set. Deletions are typically hemizygous loss of the entire gene and focal deletions primarily targeting 5’ end of the gene. Click here for file

Additional file 3 **Table S2.***RBFOX1* mRNA expression in CRC cell lines and paired CRC and normal tissues samples. Click here for file

Additional file 4 **Table S3.** Somatic mutations and single nucleotide polymorphisms found in CRC cell lines and paired CRC and normal tissues samples. Click here for file
