# Supplementary material for: Analysis of colorectal cancers in British Bangladeshi identifies early onset, frequent mucinous histotype and a high prevalence of RBFOX1 deletion
Source: Mol Cancer. 2013 Jan 3;12:1. doi: 10.1186/1476-4598-12-1 (PMC3544714; doi:10.1186/1476-4598-12-1)
Supplement: Additional file 1 — Table S1. Loss of mismatch repair proteins MLH1, MLH6 and PMS2 in a subset of British Bangladeshis colorectal cancers. No tumour showed loss of MSH2. [file 1476-4598-12-1-S1.doc]

Supplementary Table 1. Loss of mismatch repair proteins MLH1, MLH6 and PMS2 in a subset of British Bangladeshis colorectal cancers.

| *Patient* | *Age of onset* | *Mismatch Repair Protein Loss* | *Additional cancer* |
| --- | --- | --- | --- |
| E4 | 40 (early) | MLH1, PMS2 | None |
| E16 | 43 (early) | MLH1 | Gastric adenocarcinoma |
| L7 | 71 (late) | MSH6 | Lung adenocarcinoma and endometrial cancer |
| L9 | 73 (late) | MLH1, PMS2 | None |
| L14 | 65 (late) | MSH6 | None |
