# Supplementary figures and images for: Analysis of colorectal cancers in British Bangladeshi identifies early onset, frequent mucinous histotype and a high prevalence of RBFOX1 deletion
Source: Mol Cancer. 2013 Jan 3;12:1. doi: 10.1186/1476-4598-12-1 (PMC3544714; doi:10.1186/1476-4598-12-1)

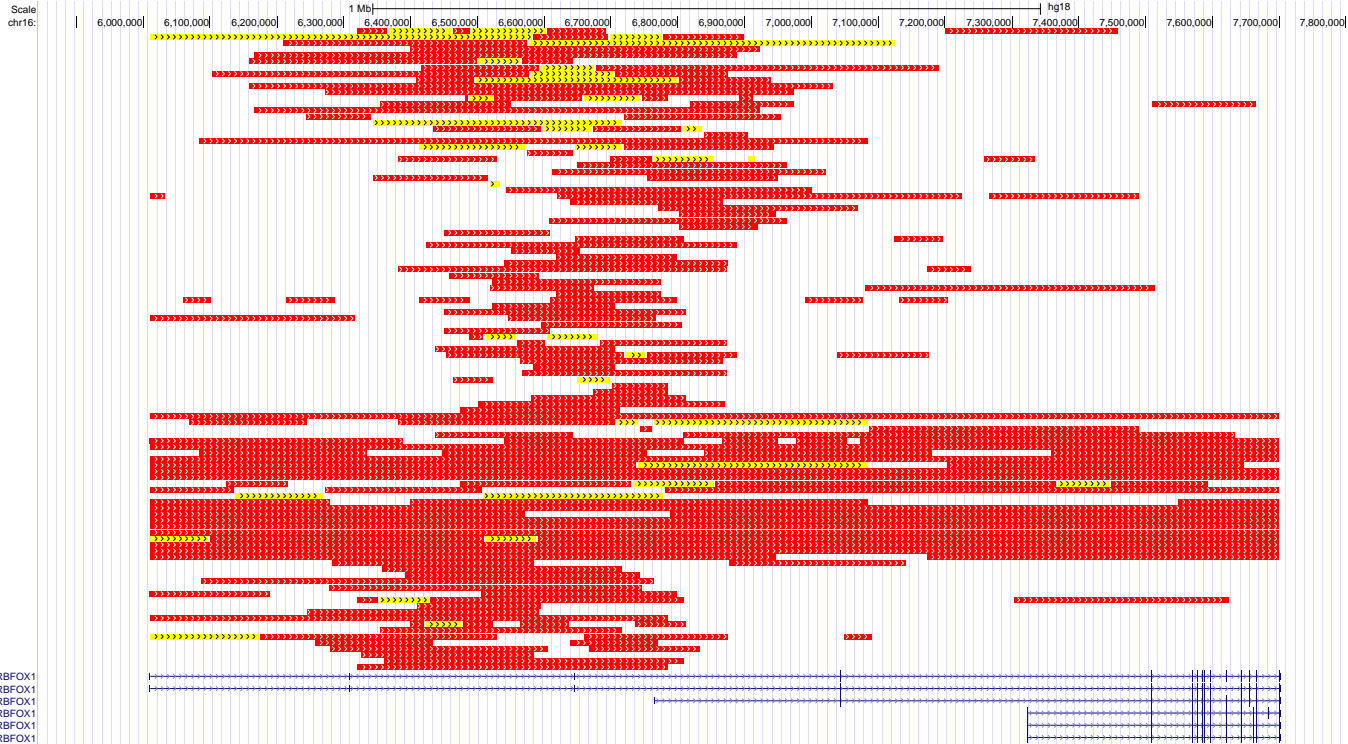

Supplement: Additional file 2 — Figure S1. UCSC Genome Browser showing hemizygous deletions/LOH (Red) and homozygous deletions (Yellow) of RBFOX1 in The Cancer Genome Atlas colorectal adenocarcinoma sample set. Deletions are typically hemizygous loss of the entire gene and focal deletions primarily targeting 5’ end of the gene. [file 1476-4598-12-1-S2.pdf]
